# Impact of the DREAMS Program on New HIV Diagnoses in Adolescent Girls and Young Women Attending Antenatal Care — Lesotho, 2015–2020

**DOI:** 10.15585/mmwr.mm7102a3

**Published:** 2022-01-14

**Authors:** Andrew R. Pelletier, Josip Derado, Limpho Maoela, Thabiso Lekhotsa, Masechache Sechache, Konosoang Nkuatsana

**Affiliations:** ^1^Division of Global HIV and TB, Center for Global Health, CDC; ^2^Elizabeth Glaser Pediatric AIDS Foundation, Washington, DC; ^3^United States Agency for International Development, Washington, DC; ^4^Lesotho Ministry of Health, Maseru, Lesotho.

Lesotho is a small, landlocked country in southern Africa with a population of approximately 2 million persons, approximately two thirds of whom live in rural areas (*1*). Lesotho has the second highest prevalence of HIV infection in the world (*2*). In 2017, 25.6% of persons aged 15–59 years living in Lesotho were HIV-positive (*3*). Strategies implemented in recent years to control HIV include efforts to reduce mother-to-child transmission and improve coverage with antiretroviral therapy, as well as increasing testing for HIV. Among persons aged 15–24 years, the HIV prevalence among females in 2017 (11.1%) was approximately three times that among males (3.4%) (*3*). The Determined, Resilient, Empowered, AIDS-Free, Mentored, and Safe (DREAMS)* program in Lesotho was started during October 2016 in two districts. DREAMS comprises a package of biomedical, behavioral, and structural interventions to address factors that make adolescent girls and young women vulnerable to HIV acquisition (*4*). The goal of the DREAMS program was to decrease HIV incidence among adolescent girls and young women by 25% after 1 year and by 40% after 2 years (*4*). After 3.5 years of program implementation in Lesotho, new HIV diagnoses among adolescent girls and young women attending antenatal care (ANC) decreased 71.4% in the two districts that implemented DREAMS compared with a reduction of 48.4% in three comparison districts without the program (p = 0.002). During 2016–2020, reductions in new HIV diagnoses among adolescent girls and young women attending ANC in Lesotho have been substantial, both in districts that have and have not implemented the DREAMS program (DREAMS and non-DREAMS districts). Apart from the DREAMS program, the decrease in new HIV diagnoses might be a result of the reduction in viral load in the population because more persons living with HIV infection became virally suppressed while on antiviral therapy, as well as other interventions such as preexposure prophylaxis, voluntary medical male circumcision, behavior change, and increased HIV diagnostic coverage.

During U.S. government fiscal years 2016–2020, the Elizabeth Glaser Pediatric AIDS Foundation was the single treatment partner funded by the U.S. President’s Emergency Plan for AIDS Relief (PEPFAR) for all districts in Lesotho (except for the first two quarters of fiscal year 2020 when mothers2mothers^†^ [M2M] replaced the Elizabeth Glaser Pediatric AIDS Foundation in two non-DREAMS districts). PEPFAR treatment partners reported quarterly on monitoring, evaluation, and reporting indicators, and provided data on the number of pregnant adolescent girls and young women who were tested for HIV at ANC by two age groups (15–24 years and ≥25 years) for each of the districts. Adolescent girls and young women were defined as females aged 15–24 years. HIV test results were categorized as negative, previously known positive, and newly test-positive. In keeping with Office of the Global AIDS Coordinator (OGAC) methodology, the new HIV diagnosis rate was calculated using the formula ([new ANC test-positives] / [total ANC clients tested − known ANC positives]) (*5*).

For this report, aggregate data for adolescent girls and young women in the two adjacent DREAMS districts (Berea and Maseru) were compared with aggregate data for adolescent girls and young women in three non-DREAMS districts (Leribe, Mafeteng, and Mohale’s Hoek). Data for women aged ≥25 years in the two DREAMS districts were also examined. Data from the first quarter of fiscal year 2016 (October 2015–December 2015) served as the baseline for comparison with the first 3.5 years of DREAMS implementation (October 2016–March 2020). A Poisson log-linear regression model was used to determine rates of decline in new HIV diagnoses among adolescent girls and young women and women aged ≥25 years attending ANC by quarter and to compare rates of decline between groups, with p<0.05 considered statistically significant. This activity was reviewed by CDC and was conducted consistent with applicable federal law and CDC policy.^§^

The number of adolescent girls and young women attending ANC in each district varied little from year to year (Table). Among adolescent girls and young women in the two DREAMS districts, the percentage of new HIV diagnoses decreased from 11.4% in the first quarter of fiscal year 2016 to 3.3% in the second quarter of fiscal year 2020, for a total reduction of 71.4% (p<0.001) (Figure). A decline of 48.4% occurred among adolescent girls and young women in the three non-DREAMS districts, from 7.7% to 4.0% (p<0.001). The difference in the percentage reduction among adolescent girls and young women in DREAMS versus non-DREAMS districts was statistically significant (p = 0.002). When restricting the analysis to women aged ≥25 years in the two DREAMS districts, the percentage new HIV diagnoses decreased 54.6%, from 16.6% to 7.5% (p<0.001).

**TABLE:** Number and percentage of new HIV diagnoses among adolescent girls and young women attending antenatal care, by district DREAMS implementation status and age group — Lesotho, fiscal years 2016–2020

Fiscal year and quarter	Females aged 15–24 years attending antenatal care				Women aged ≥25 years attending antenatal care	
	DREAMS districts*		non-DREAMS districts^†^		DREAMS districts*	
	No. of new HIV diagnoses (%)	No. tested	No. of new HIV diagnoses (%)	No. tested	No. of new HIV diagnoses (%)	No. tested
FY2016 Q1	158 (11.4)	1,392	93 (7.7)	1,215	171 (16.6)	1,033
FY2016 Q2	153 (9.4)	1,623	111 (7.3)	1,516	205 (16.2)	1,267
FY2016 Q3	155 (9.0)	1,715	100 (6.7)	1,489	183 (16.0)	1,147
FY2016 Q4	130 (7.9)	1,647	76 (5.7)	1,341	157 (12.9)	1,214
FY2017 Q1	105 (7.3)	1,444	66 (6.2)	1,063	138 (13.8)	1,003
FY2017 Q2	144 (8.3)	1,741	83 (5.7)	1,450	150 (12.0)	1,253
FY2017 Q3	112 (7.3)	1,531	73 (5.4)	1,358	108 (10.4)	1,041
FY2017 Q4	101 (6.5)	1,545	74 (5.2)	1,415	119 (9.8)	1,217
FY2018 Q1	80 (5.9)	1,346	56 (5.2)	1,087	117 (12.0)	976
FY2018 Q2	107 (5.9)	1,823	60 (4.2)	1,419	137 (11.2)	1,228
FY2018 Q3	95 (5.6)	1,703	55 (3.8)	1,466	120 (10.4)	1,152
FY2018 Q4	106 (6.1)	1,745	68 (5.1)	1,335	137 (11.0)	1,242
FY2019 Q1	69 (4.4)	1,553	56 (4.8)	1,179	109 (9.7)	1,126
FY2019 Q2	92 (5.0)	1,837	60 (4.1)	1,454	131 (9.3)	1,403
FY2019 Q3	81 (4.9)	1,657	48(3.6)	1,340	109 (9.1)	1,198
FY2019 Q4	64 (3.8)	1,679	50 (3.6)	1,377	104 (8.4)	1,233
FY2020 Q1	53 (3.7)	1,437	44 (4.0)	1,109	73 (7.3)	994
FY2020 Q2	56 (3.3)	1,723	55 (4.0)	1,392	101 (7.5)	1,344

**Abbreviations**: DREAMS = Determined, Resilient, Empowered, AIDS-Free, Mentored and Safe; FY = fiscal year; Q = quarter.

* Berea and Maseru.

^†^ Leribe, Mafeteng, and Mohale’s Hoek.

**FIGURE:**
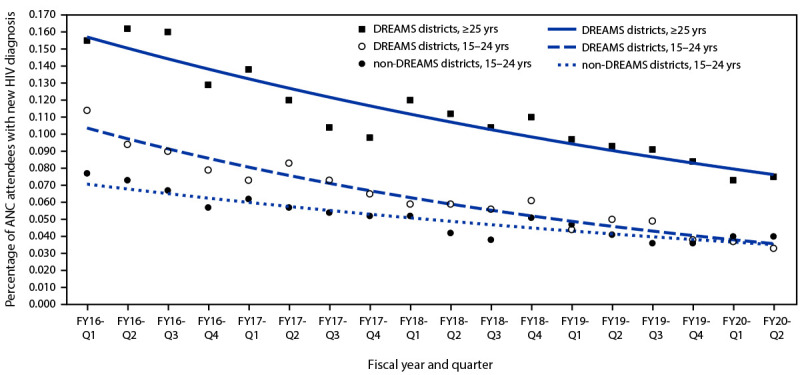
New HIV diagnoses* among adolescent girls and young women attending antenatal care, by district DREAMS implementation status and age group — Lesotho, fiscal years 2016–2020 **Abbreviations**: ANC = antenatal care; DREAMS = Determined, Resilient, Empowered, AIDS-Free, Mentored, and Safe; FY = fiscal year; Q = quarter. * Trend lines derived from Poisson regression model.

## Discussion

Results of the first 3.5 years of the DREAMS program implementation in Lesotho showed a substantial reduction in new diagnoses of HIV infection among adolescent girls and young women in both DREAMS and three non-DREAMS districts. This occurred at a time when the national HIV treatment program in Lesotho had begun universal initiation of antiretroviral therapy for all HIV-positive persons regardless of CD4 count, and the country had demonstrated good progress toward the United Nations Programme on HIV and AIDS (UNAIDS) 90–90–90 goals (90% of persons living with HIV know their status, 90% of those with HIV who know their status are receiving treatment, and 90% of those receiving treatment are virally suppressed) (*6*). The Lesotho Population-Based HIV Impact Assessment (LePHIA), conducted during November 2016–May 2017, found that 81% of persons living with HIV infection knew their status, 92% of those who knew their status were on treatment, and 88% of those on treatment were virally suppressed (*3*). However, the results for adolescent girls and young women (61%–90%–76%) were cause for concern (*7*).

The concurrent reduction in new HIV diagnoses among adolescent girls and young women attending ANC in two DREAMS and three non-DREAMS comparison districts might have resulted from an overall reduction in viral load in the population because more persons living with HIV infection became virally suppressed. Other factors, including preexposure prophylaxis, voluntary medical male circumcision, behavior change, and increased HIV diagnostic coverage might have also played a role. The reductions in new HIV diagnoses at ANC among adolescent girls and young women in the three non-DREAMS comparison districts and among women aged ≥25 years in the two DREAMS districts suggests that much of the decline was attributable to factors other than DREAMS, because these women were not eligible for the program, either because of where they lived or their age.

The findings in this report are subject to at least five limitations. First, changes in new HIV diagnoses among adolescent girls and young women attending ANC might be an inaccurate measure of changes in HIV incidence. Reporting of new HIV diagnoses could be affected by multiple factors related to surveillance efforts (e.g., nonuniversal ANC attendance and incomplete HIV testing during ANC). However, the LePHIA indicated that 97.1% of pregnant women aged 15–49 years had attended ANC at least once, and 95.6% knew their HIV status (*3*). Second, data for HIV testing at ANC were obtained from implementing partners rather than from the database maintained by OGAC. This was done because the Elizabeth Glaser Pediatric AIDS Foundation data were more complete and contained fewer values considered to be outliers. Third, the five districts (including the two DREAMS and three non-DREAMS districts) might differ in ways that affect a direct comparison between DREAMS and non-DREAMS districts. However, the population of Lesotho is largely homogenous, and there are few obvious differences among the five districts where 73% of the population resides (*1*). Fourth, this assessment was based on an ecologic analysis. Data were not available for individual adolescent girls and young women in DREAMS districts to compare outcomes of those participating in the program with those of nonparticipants. Data were also not available to assess what percentage of the eligible population received a complete suite of program services. Finally, data from Lesotho might not be representative of data from the other nine countries in Africa that were part of the original DREAMS program.

The second LePHIA was completed in March 2020. Although HIV incidence has declined among persons aged ≥15 years, marked disparities still exist in incidence and prevalence between women and men (*8*). LePHIA was not designed to provide a specific incidence estimate for adolescent girls and young women at the district level. Therefore, LePHIA results cannot be used to directly assess the impact of the DREAMS program in Lesotho. However, the results of the second LePHIA indicate that substantial work remains to address gender disparities. Conducting similar analyses of ANC data in other countries implementing DREAMS could determine whether the results from Lesotho are generalizable and complement the findings of a recent evaluation of DREAMS on HIV incidence among adolescent girls and young women in Kenya and South Africa (*9*). Results of these studies might better clarify the impact of DREAMS and help guide future decisions on how best to reduce HIV incidence among adolescent girls and young women in Africa.

**Summary**

**What is already known about this topic?**

During 2016–2017, HIV prevalence among adolescent girls and young women in Lesotho was approximately three times that among young men. The Determined, Resilient, Empowered, AIDS-Free, Mentored, and Safe (DREAMS) program was established in 2016 to decrease HIV incidence among young women.

**What is added by this report?**

New HIV diagnoses among adolescent girls and young women attending antenatal care decreased significantly in both DREAMS and non-DREAMS districts, although reductions were greater in DREAMS districts.

**What are the implications for public health practice?**

Apart from DREAMS, the decrease in new HIV diagnoses might be a result of the reduction in viral load in the population because more persons living with HIV infection became virally suppressed while on antiviral therapy, preexposure prophylaxis, voluntary medical male circumcision, behavior change, and increased HIV diagnostic coverage.

## Footnotes

* https://www.state.gov/pepfar-dreams-partnership/

†
https://m2m.org/

§ 45 C.F.R. part 46; 21 C.F.R. part 56; 42 U.S.C. Sect. 241(d), 5 U.S.C. Sect. 552a, 44 U.S.C. Sect. 3501 et seq.
